# Effect of app-based mindfulness on extinction recall – a 7T-fMRI study

**DOI:** 10.1038/s41598-026-45569-z

**Published:** 2026-03-25

**Authors:** Johannes Björkstrand, Emil Olsson, Oisin Hugh Clancy, Stefan Möller, Isabella M. Björkman-Burtscher, Nishika Raheja, David Sjöström, Marino Persiani, Walter Staiano, Ulrich Kirk

**Affiliations:** 1 Department of Psychology, Lund University, Lund, Sweden; 2 Department of Radiology, Institute of Clinical Sciences, Sahlgrenska Academy, University of Gothenburg, Gothenburg, Sweden; 3 Department of Radiology, Sahlgrenska University Hospital, Region Västra Götaland, Gothenburg, Sweden; 4 Fralin Biomedical Research Institute at VTC, Virginia Tech, Roanoke, VA USA; 5 Child & Adolescent Psychiatry, Lund University, Lund, Sweden; 6 Department of Psychology, University of Southern Denmark, Odense, Denmark; 7 Department of Physical Education and Sport, University of Valencia, Valencia, 46010 Spain

**Keywords:** Neuroscience, Psychology, Psychology

## Abstract

Fear-based disorders affect millions worldwide, yet current treatments show limited effectiveness for many patients. While mindfulness is increasingly used clinically for anxiety and trauma disorders, the neural mechanisms underlying its effects on fear processing remain unclear. We conducted a randomized controlled trial using 7T fMRI to test whether mindfulness training enhances fear extinction recall—a process critical for recovery from these disorders. Healthy participants received four weeks of app-based mindfulness meditation (*n* = 27) or served as waitlist controls (*n* = 28), then underwent fear conditioning and extinction recall testing. Mindfulness training specifically enhanced extinction recall, reducing threat responses to extinguished cues by both physiological (skin conductance, *p*=.028) and neural measures. Critically, our findings reveal a candidate mechanism: mindfulness reduced activation in subcortical threat-processing regions (amygdala, striatum, supplementary motor area) without enhancing cognitive control areas, a pattern consistent with direct modulation of fear circuits rather than top-down inhibition, though top-down contributions cannot be excluded. This pattern is consistent with mindfulness enhancing safety memory retrieval through implicit rather than explicit emotion regulation, providing preliminary neurobiological evidence relevant to optimizing mindfulness-based treatments. Our findings suggest that mindfulness training, whether administered before or alongside exposure therapy, could potentially enhance therapeutic outcomes by improving the consolidation and retrieval of safety memories, although replication in larger clinical samples is needed.

## Introduction

Over the last few decades, mindfulness-based practices have increased in popularity in the western world because of its positive effects on psychological functioning^1^. Several studies show decreases in negative affective states such as stress, anxiety and depression as a result of mindfulness practice both in clinical and non-clinical populations^2–4^. In particular, mindfulness-based interventions have shown promise for anxiety disorders, with meta-analytic evidence supporting their efficacy across generalized anxiety disorder, social anxiety disorder, and PTSD^5,2,6^. These clinical effects are thought to be mediated by changes in attention regulation, body awareness, emotion regulation, and changes in self-perspective^7^. Importantly, recent behavioral research has begun to connect these clinical outcomes to fear conditioning and extinction processes, suggesting that the therapeutic effects of mindfulness on anxiety may be partly explained by enhanced extinction learning and recall (Treanor, 2011;^8^. However, the psychological and neurobiological mechanisms through which mindfulness achieves its effects on negative affective states are still unclear and under much debate^9,10^. Generally, mindfulness has been shown to improve emotion regulation^11^, which could be dependent both on explicit emotion regulation strategies and implicit processes related to expression of negative emotional states, such as fear and anxiety^12^. Explicitly and purposely adopting an accepting and non-judgmental mindset to internal emotional experiences may well decrease the subjective impact of negative emotional reactions^13,14^ facilitating adaptive behavioral strategies in anxiety-provoking situations and could be considered a form of cognitive reappraisal. This type of emotion regulation is held to be dependent on prefrontal and parietal cortical regions, belonging to the cognitive-control network, exerting inhibitory control over limbic regions^15,16^, which is effective but also dependent on conscious cognitive effort^12^. However, mindfulness could also potentially influence emotional reactions through implicit emotion regulation. One proposed pathway for such implicit regulation involves alterations in extinction learning, although implicit regulation may also operate through other mechanisms such as attentional deployment^8,17–19^; Treanor,^20^.

Extinction occurs when a previously conditioned stimulus (CS) is repeatedly presented without its associated outcome^21,22^, for example when a cue (such as a tone or image) that has previously been paired with painful stimulation (such as an electrical shock), is presented without this outcome and the CS loses its predictive value. Conditioning and extinction are ongoing learning processes that continuously shape our emotional reactions to cues we encounter in our daily lives as we establish and update emotional associations to things and situations that we are confronted with^23,24^. These processes are also held to be highly relevant for anxiety- and fear-related mental disorders, which are established and maintained through emotional learning processes related to conditioning and extinction^25,26^. The neurobiological processes that enable this type of emotional learning have been extensively studied in animals^27,28^ and humans^29,30^ through the use of pavlovian fear/threat conditioning protocols and subsequent extinction^31^. In fMRI-studies on humans, a “fear network” has been identified as a group of widely distributed brain regions that are commonly activated to mediate learned fear responses. This network is comprised of the bilateral anterior insular cortex (AIC), amygdala, orbitofrontal cortex (OFC), substantia nigra, ventral striatum, thalamic nuclei, and dorsal anterior cingulate cortex (dACC) (for full review see^32^. In conditions such as post-traumatic stress disorder (PTSD) and anxiety disorders where major symptoms are rooted in maladaptive fear learning or extinction, these abnormalities have been linked to the “fear network”^33^. Extinction of fear reactions is similarly dependent on this network^32^, but also entails other regions. The ventromedial prefrontal cortex (vmPFC) and hippocampus are identified as particularly important in extinction learning^34,35^, where vmPFC exerts inhibitory control over limbic regions that drive fear expression, such as the central amygdala^34,35^, and hippocampus encodes contextual aspects of extinction learning, to control in which contexts fear reactions should be expressed or inhibited^34,35^. While previous neuroimaging studies have proposed mechanisms by which mindfulness may influence extinction processes, including hippocampal contributions to extinction recall^36^, the specific neural pathways remain to be fully elucidated. Potential effects could occur through enhanced top-down inhibitory control, through direct modulation of threat-processing regions, or through a combination of these pathways. Notably, extinction is not considered to constitute an undoing of the original memory trace but rather is a form of safety-learning where a separate memory trace is established, which then suppresses the expression of the “fear/threat” memory, at least in contexts that after extinction are designated as safe (i.e. the extinction context) (Bouton,^37^. As such, extinction memories appear less robust compared to fear memories. Even after successful extinction, where fear responses are diminished after multiple non-reinforced presentations of conditioned cues, these responses tend to return either after a switch in context (called renewal), or simply after the passage of time (called spontaneous recovery)^38^. This particular phenomenon, often referred to as extinction recall, has received a lot of attention in research related to fear and anxiety^39,40^. First-line treatments for fear-related disorders are cognitive behavioral therapy^41,42^ where exposure to anxiety provoking stimuli is a crucial part of treatment^43,44^. From a theoretical standpoint, exposure-based treatments build on inducing extinction learning^45,46^, and although these treatments are effective^47^, not all patients respond effectively to these treatments^48,49^. A major theme in psychiatric translational research has been to uncover ways of facilitating extinction recall, and thus improve treatment outcomes for these disorders^50^ for example by exploiting crucial time windows related to memory reconsolidation^51,52^ using drugs that boost synaptic plasticity^53,54^, or employing psychological interventions such as mindfulness^6,55^.

Multiple review articles concerning the psychological and neurobiological mechanisms of mindfulness have put forward the hypothesis that the effect of mindfulness on negative affective states, particularly fear and anxiety, could be dependent on alterations in extinction learning and the subsequent retrieval of safety memories^19^; Treanor,^56^. This is based on the supposition that mindfulness can facilitate extinction learning, through multiple potential pathways: first, through enhanced top-down cognitive control mechanisms involving prefrontal regions; second, through direct modulation of subcortical threat-processing circuits; or third, through improved attention to safety cues and interoceptive experiences combined with acceptance of negative emotional states, thereby enhancing the encoding and retrieval of inhibitory safety memories^19^; Treanor,^56^. The hypothesis is also supported by neuroimaging studies showing an association between the practice of mindfulness, and functional as well as structural alterations in brain regions known to be involved in threat processing and extinction, such as the amygdala, hippocampus, insular cortex, and the PFC^57,9,19^, including studies that directly investigate the effect of mindfulness on processing of negative emotions^58,59^. Conceivably, continuous mindfulness practice induces plasticity in these regions, which then alters signaling in neural pathways that handle threat processing, thereby influencing the encoding, consolidation and retrieval of safety memories. This would further suggest that mindfulness can alter implicit aspects of emotional processing, and that conscious cognitive effort (i.e. purposely adopting an accepting and non-judgmental mindset to inner experiences) during negative affective states is not always essential, in order for mindfulness practice to reduce negative affect. However, concrete empirical support for this hypothesis is still lacking since very few studies have directly investigated the effect of mindfulness on fear extinction, and the neural regions involved in these processes remain to be elucidated.

We have previously shown that 30 days of daily mindfulness reduces fear expression (skin conductance responses to conditioned cues) during early extinction recall, as compared to a non-treatment control group^8^. This effect was specific to extinction recall, since no group differences were observed either during fear conditioning, or extinction phase. Two neuroimaging studies have similarly investigated the hypothesis that mindfulness affects extinction recall^17,36^. However, both of these studies were unable to demonstrate an effect of mindfulness intervention on fear expression as measured by skin conductance responses. While Sevinc et al.,^36^ had neuroimaging data from a larger sample (42 MBSR and 25 controls), the skin conductance analysis that could demonstrate behavioral effects was limited to a subset of participants (8 vs. 8) due to the exclusion of participants who did not exhibit signal changes during conditioning. Similarly, Hölzel et al.,^17^ had a small final sample for their fear conditioning analysis (11 vs. 8). Thus, the absence of a behavioral effect on fear expression in these studies could be due to insufficient statistical power in the skin conductance analyses.

In this study, we investigated the effect of mindfulness training on fear learning using a readily accessible 30-day app-based mindfulness intervention and a two-day extinction and recall protocol. In this fear conditioning paradigm, visual stimuli were paired with aversive stimuli in the form of benign electrical shocks, followed by immediate extinction, and 24 h later, extinction recall was evaluated. Based on previous findings^8^, we hypothesized that the group trained in mindfulness would exhibit higher extinction recall of threat responses as compared to the waitlist control group assessed through skin conductance responses (SCRs). Given that the fear network, as well as the inhibitory regions, vmPFC and hippocampus, are expected to be implicated in the extinction, we paired this with state-of-the-art 7T fMRI brain imaging to investigate the effect of the mindfulness intervention on these neural regions during this fear activation. Understanding how this network responds to fear-mitigating interventions such as mindfulness is crucial for understanding the etiology of these conditions as well as the underlying mechanisms of treatment and recovery.

## Methods

### Participants

Participants were recruited through advertisement on social media and word of mouth. After signing up using an online questionnaire, they were subsequently screened for suitability via telephone interview. Participants were required to be in overall good health and only have limited experience with mindfulness. Exclusion criteria were ongoing diagnosed mental or neurological disorder, ongoing treatment for mental or neurological disorder, and contraindications for undergoing MRI.

In total 76 participants were enrolled in the study. 13 participants dropped out before the first examination due to personal reasons and scheduling difficulties. Additionally, 8 participants did not complete the experimental session on day 2 due to scanner malfunction and were excluded. Of the remaining 55 participants, all had complete skin-conductance-data for day 2 and were included in the final analysis, but one additional subject had missing skin-conductance-data for day 1 due to technical errors and was excluded from analysis pertaining to day 1. For fMRI-data day 2, investigating extinction recall, 5 additional subjects had missing data due to technical or clerical errors, and one subject was excluded due to motion artifacts. We included as many subjects as possible in each analysis and thus the sample-size for day 1 skin-conductance analysis is *n* = 54 (Mindfulness [MF]: *n* = 27; Waitlist Control [WL]: *n* = 27), for day 2 skin-conductance analysis is *n* = 55 (MF: *n* = 27; WL: *n* = 28), and for fMRI analysis day 2 is *n* = 49 (MF: *n* = 25; WL: *n* = 24). For self-report outcomes 53 participants had complete data (MF *n* = 26; WL *n* = 27).

The final sample (*n* = 55) had a fairly even gender distribution (60% female) which did not differ between groups (chi2 = 1.47; df = 1; *p*=.226; MF: 52% female; WL: 68% female). Mean age was 26.2 years (SD = 7.7; Min = 19; Max = 56), and was similar in both groups (MF: M = 26.1; SD = 7.3; WL: M = 26.3 ; SD = 8.3) with no differences (t(53) = 0.07; *p* = .948).

**Fig. 1:**
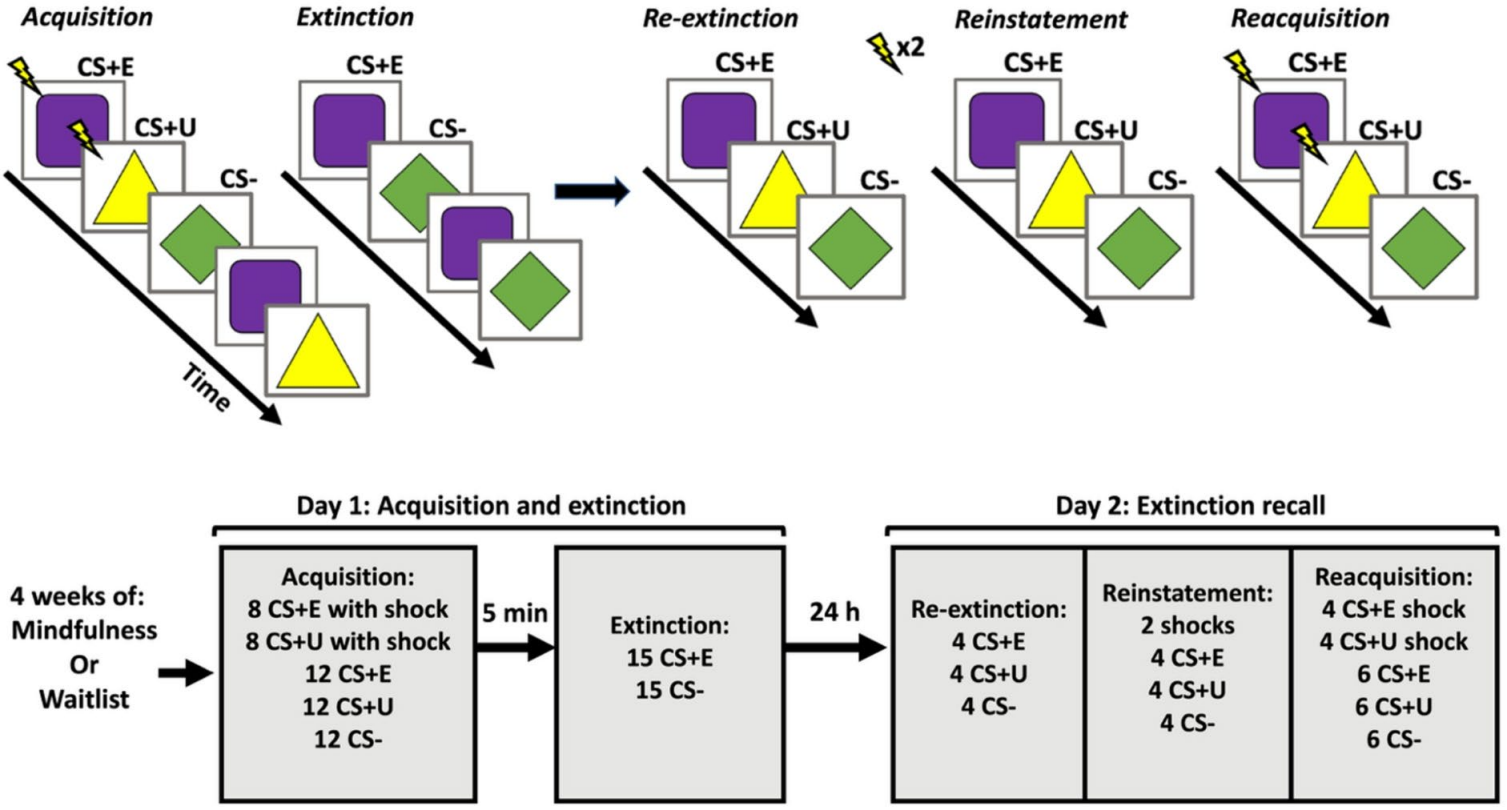
Overview of design and experimental protocol. Participants were assigned to either four weeks of daily mindfulness using a digital app with guided exercises, or to a waitlist control group. After four weeks, participants underwent a two-day experimental protocol including acquisition and extinction of conditioned threat responses on day 1 and a test of extinction recall on day 2. During acquisition, two neutral cues were paired with an aversive shock (CS + E; CS + U), whereas a third cue was presented, but never followed by a shock (CS-), serving as a control stimulus. This was followed by extinction where one of the shock-paired cues (CS + E) was repeatedly presented without shock, along with additional presentation of the CS-. On day 2, extinction recall was evaluated using a testing protocol where all three cues (CS + E, CS + U and CS-) were presented again. The testing protocol consisted of three consecutive phases; re-extinction (4 non-reinforced presentation of each CS), re-instatement (2 unsignaled shocks followed by 4 non-reinforced CS presentations), and reacquisition (4 reinforced presentation of each CS+, interspersed with 6 non-reinforced presentations of each CS).

The study was approved by the Swedish National Ethical Review Authority (registration number: 2019 − 00204) and conducted in line with the Helsinki declaration. All subjects provided written informed consent before entering the study and received 500 SEK for participation as outlined to the Swedish National Ethical Review Authority (Fig. 1).

### Design

The study was a non-blinded randomized between-group design with a wait-list control group. Participants with little previous experience with mindfulness were recruited and randomly assigned to receive either four weeks of daily mindfulness (approx. 15 min/day) using guided mindfulness exercises provided through a commercial and easily implementable mindfulness-app (headspace.com) and the control group was assigned to a 4-week wait-period, and instructed not to engage in mindfulness practices during this period. Subsequently, participants underwent a 2-day experimental protocol consisting of fear conditioning and immediate extinction on day 1, and a test for extinction recall on day 2. Both sessions took place in an MRI-scanner, and psychophysiology (skin conductance) and functional neuroimaging (fMRI) data were obtained for all three experimental phases.

Main outcomes of interest were event-related skin conductance responses (SCRs) and fMRI-BOLD responses to conditioned stimuli during day 2, since we have shown effects specifically on extinction recall in a previous study using a similar design^8^. Secondary outcomes included event-related SCRs to conditioned stimuli during day 1 to confirm successful acquisition and extinction of threat responses, user-data logged in the mindfulness-app to confirm intervention compliance in the intervention group, as well as self-report data collected pre- and post-intervention (trait-mindfulness as well as symptoms of depression and anxiety).

### Procedure

After inclusion criteria were met, participants provided written informed consent and completed an online-questionnaire assessing trait-mindfulness and current level of anxiety and depression symptoms. Subsequently, they were randomized to either receiving four weeks of daily mindfulness training or assigned to a four-week waitlist condition. The mindfulness group were then given access to a commercial mindfulness app (headspace.com) and instructed to engage in daily mindfulness using the guided meditation modules provided in the app. They were further instructed to focus on the basic programs, levels 1–3 on the app and aim for 10 min/day for the first 8–10 days, then increasing to 15 min/day after 8–10 days and finally to 20 min/day for the remainder of the study period. Beyond the instructional content in the app and directions on how to access this material, no further instruction in mindfulness was provided. The control group was instructed that they should not engage in mindfulness or any other meditation practice for the duration of the study, but that they would get access to the same material after their participation ended. Following this four-week period, participants in both groups were then scheduled for the experimental examinations for the implementation of the 2-day extinction and recall protocol.

Experimental sessions took place at the Lund Biological Imaging Center, National 7T Facility, over two consecutive days, including fear acquisition and extinction on day 1, and a test for extinction recall on day 2 approximately 24 h. later. After the second testing session, participants again completed the online questionnaires on mindfulness, depression and anxiety.

### Experimental protocols

On day 1, participants first underwent fear conditioning followed by extinction. During fear acquisition participants were shown three different geometrical figures (purple square with rounded edges; yellow triangle and green square diamond) two of which were paired with mild electrical shocks. One of the two shock-paired cues subsequently underwent extinction (designated CS + E), whereas the other did not (designated CS + U), and the un-paired cue (designated CS-) thus signaled safety and functioned as a control-stimulus. The geometrical figures assigned as CS + E, CS + U and CS- were counterbalanced across subjects. The acquisition phase included 8 reinforced presentations and 12 non-reinforced presentations of each CS + as well as 12 non-reinforced presentations of the CS-, resulting in a reinforcement-rate of 40%. Each stimulus was presented for 4s, and for reinforced trials, co-terminated with shock delivery with a duration of 250ms. Each stimulus presentation with an intertrial interval of fixation-cross on a white background randomly jittered between 7 and 13 s. Presentation order was randomized with the following restrictions: acquisition always began with a reinforced presentation of each CS+; each reinforced trail was followed by either one or two non-reinforced presentation of each CS; and that for non-reinforced trials, all three CS had to be presented before starting a new presentation cycle. This procedure aimed at ensuring even distribution of reinforced and non-reinforced trials across the testing run and counteracted order effects such as multiple sequential repetitions of the same stimulus. Before each session began, participants only received the following instruction: *“During this task you will see different geometrical figures appear on the screen. All you have to do is to look at the pictures. Throughout the task you may receive electrical shocks from time to time.*” Thus, participants received no information about picture-shock contingency beforehand, nor were they instructed to apply any meditation techniques during the experiment.

The extinction phase followed the acquisition after approximately 5 min delay. Extinction consisted of 15 non-reinforced presentations each of the CS + E and CS-. Each stimulus was presented for 4s with an intertrial interval of fixation cross on a white background randomly jittered between 7 and 13 s. Presentation order was randomized with the restriction that both CS had to be presented once before starting a new presentation cycle.

After 24 h, participants underwent a protocol investigating return of conditioned threat responses. This protocol consisted of three phases: re-extinction, reinstatement and reacquisition. The re-extinction phase included 4 non-reinforced presentations each of CS + E, CS + U and the CS-. This was followed by reinstatement, which included two unsignaled shocks, followed by another 4 non-reinforced presentations of each CS. Finally, subjects underwent reacquisition, where the CS + E and CS + U, again were paired with shocks. This phase was identical to the initial acquisition protocol, but with half as many trials, i.e. 4 reinforced presentations of the CS + E and CS + U respectively, and 6 non-reinforced presentations of all CS. These three phases were delivered in one continuous run. Similar to previous experimental phases, stimuli were presented for 4s with an intertrial interval of fixation cross on a white background randomly jittered 7–13 s. During the re-extinction and reinstatement phase, presentation order was randomized, with the restriction that all three CS had to be presented once before starting a new presentation cycle. Presentation order during reacquisition followed the same pattern as during day 1. All the tasks were programmed and delivered using E-prime 2.0 (Psychology Software Tools, Pittsburgh, PA).

### Self-report questionnaires

To evaluate potential pre-existing group differences as well as intervention effects - in trait mindfulness, anxiety, and depression - participants completed self-report questionnaires before and after the study. This included the Mindful Attention Awareness Scale (MAAS)^60^, the Generalized Anxiety Disorder-7 (GAD-7)^61^, and the Montgomery-Åsberg Depression Rating Scale (MADRS)^62^. These are widely used and previously validated questionnaires for the measurement of trait mindfulness, current anxiety symptoms and current depression symptoms, respectively.

### Skin conductance and pain stimulation

Electric shocks were used as unconditioned stimuli (US) and delivered to the lateral surface of the lower right leg using disposable radiotransluscent electrodes (EL509, BIOPAC Systems, Goleta, CA, USA) with a STM100C module connected to the STM200 constant voltage stimulator and controlled by the BIOPAC MP150 (BIOPAC Systems, Goleta, CA, USA). Participants determined the strength of the electric shock through a staircase procedure with the instruction that the shock should be unpleasant, but at the same time, endurable for the duration of the experiment. Since US-level was set individually for each participant using a work-up procedure, this could introduce systematic differences between groups. To evaluate this possibility, data for the individual shock-level set by each participant were extracted and compared across groups. The average US-intensity was 48.9 volts (SD = 13.4 volts; Min = 27.5 volts; Max = 90 volts), highly similar for both groups (MF: Mean = 50.0 volts; SD = 15.4 volts; WL: Mean = 47.9 volts; SD = 11.4 volts), with no group differences (t(52) = 0.57; *p* = .569), indicating that US-level was not a potential confound for the study outcomes.

To quantify changes in physiological arousal to conditioned stimuli, event-related skin conductance responses (SCRs) were measured using two Ag–AgCl electrodes (EL509 Biopac electrodes) filled with isotonic electrode gel (GEL101 Biopac gel) attached to the palmar surface of the hypothenar eminence of the left hand. SCRs were amplified and recorded with a sampling rate of 1000 Hz using the Biopac MP150 system and Acqknowledge software, version 4.2 (BIOPAC Systems, Goleta, CA, USA). Subsequently, a low-pass filter of 0.5 Hz was applied to the skin conductance signal in order to filter out high frequency artefacts. Then, similar to previous studies^63–66^, event-related SCRs were extracted using a local baseline approach, by deducting the mean value of the skin conductance signal 1.2–1.5 s after stimulus onset from the peak value 1.5–4.5 s after stimulus onset. Subsequently, SCRs were square root transformed and then range-corrected by dividing all values by each participant’s maximum SCR (in all cases a US-response), resulting in SCRs ranging from 0 to 1. This minimizes the influence of individual differences and isolates the experimental effects^67^.

Skin conductance analysis: For SCRs only non-reinforced trials were used for analysis to avoid possible US-contamination. To reduce intra-individual variability, responses were averaged in to bins. For fear conditioning responses were averaged over three bins of four trials each (early; mid; late), for extinction responses were averaged over three bins of 5 trials (early, mid, late), and for the extinction recall test responses were averaged over each experimental phase, i.e. re-extinction (4 trials), reinstatement (4 trials) and reacquisition (6 trials). Outcomes were investigated with repeated measures analysis of variance (rm-ANOVAs) implemented in JASP (version 0.18.3). Greenhouse-Geisser correction was applied where appropriate.

### Brain imaging

Patient preparation: After arriving at the MR-facility, the subjects were asked to change into hospital scrubs, and were screened for contraindications of undergoing MR. They were informed of the scanning and task procedures and were then placed in the scanner after being fitted with earplugs and sound-insulating clay to reduce noise from the scanner. Participants were placed in supine position on the scanner table with their heads placed in a padded head-coil equipped with a slanted mirror allowing the subject to see a projected screen at the opposite end of the scanner bore where task instructions and stimuli were shown. Due to the close fit of the head-coil no additional head-fixation was deemed necessary except for adjusting the thickness of the padding as needed. Participants were equipped with an alarm-button which the subject were told to press if they needed to interrupt an ongoing examination.

Data were acquired using a 7T whole body MR scanner (Philips Achieva, Philips, Best, The Netherlands) with a head coil with 32 recieve and 2 transmit channels (Nova Medical, Wilmington, MA). The scanning procedure consisted of a survey-scan, a B0-field map to allow for adjustment of shimming parameters, a T1-anatomical scan and on day 1 two functional scans (fear conditioning and extinction tasks) and on day 2 one functional scan (fear retention test). Acquisition time was approximately 35–45 min for each session.

Anatomical 3D T1-weighted images were acquired using a MPRAGE sequence with the following parameters: repetition time (TR) = 5.0 ms, cycle duration=3500ms, inversion time (TI) = 1200 ms, flip angle = 6°, acquired voxel size = 1.0 × 1.0 × 0.5 mm3, reconstructed voxel size 0.87 mm isotropic, SENSE acceleration factor 2 × 2. BOLD fMRI was collected using a simultaneous multi-slice echo-planar imaging (SMS-EPI) sequence with the following parameters: TR = 1500 ms, TE = 25 ms, flip angle 55°, voxel size = 2 × 2 × 2 mm^3^, slice gap = 0.2 mm, slices = 50, multi-slice acceleration factor = 2, SENSE acceleration factor = 3. The field of view encompassed the entire brain, excluding the lower half of the cerebellum.

Preprocessing and noise removal was achieved using the CONN toolbox^68^ in SPM12 (fil.ion.ucl.ac.uk/spm/software/spm12). For preprocessing, the default pre-processing pipeline was used, omitting the slice-time correction step in light of the short TR. Thus, preprocessing included realignment and unwarping of the functional images, co-registration of the functional images to the T1-weighted anatomical image, outlier detection using the artefact removal tool (cut-off: 0.9 mm frame-wise displacement; 5SD deviation from global BOLD signal), segmentation and normalization to standard MNI-space (2 mm isotropic voxels) by applying the transformation parameters from the segmentation of the T1-weighted image, and spatial smoothing of the functional images using a 6 mm FWHM Gaussian filter. Then, functional images were denoised using the CONN-toolbox denoising pipeline, to remove effects of movement, physiological noise and other artefacts. This denoising method uses linear regression and several covariates of no interest to remove this noise-related variability from the fMRI-BOLD time-series. This includes BOLD-signal extracted from white-matter (WM) and cerebrospinal fluid (CSF), i.e. anatomical component-based noise correction (aCompCor), subject movement parameters, and outlier scans identified during preprocessing, i.e. scrubbing^69^. For denoising we included 10 noise components for aCompCor including global signal and the first four components resulting from principal component analysis of the WM and CSF signal respectively, 12 noise components related to subject movement, i.e. 3 translational and 3 rotational parameters and their first-order derivatives, and finally outlier scans with one noise components added for each volume considered an outlier. This value varied between participants and a cut-off was set that any run missing more than 10% of volumes would be excluded. One subject in the control group was excluded due to the number of missing volumes exceeding 10%. Finally, a high-pass filter of 0.008 Hz was applied to the signal in order to eliminate very slow signal fluctuations.

fMRI analysis: For each participant, images from the functional scan on day 2 was entered into a single first-level model. The model included regressors (onset time and duration) for non-reinforced presentations the CS + E, CS + U and CS- respectively, separated by task phase, i.e. re-extinction (trial 1–4), reinstatement (trial 5–8), and reacquisition (trial 9–14). Additionally, separate regressors for the reinforced presentations of CS + E and CS + U during reacquisition were added, as well as a regressor for US delivery during reinstatement and reacquisition, modeled as a stick function with 0 s duration. Each of these regressors were then convolved with the canonical hemodynamic response-function. Since spurious activity related to subject motion had already been accounted for during denoising, no realignment parameters were added to first-level models. To examine activation patterns during the task, contrast images for each participant were created comparing CS + E and CS + U to the CS- for all experimental phases separately, i.e. re-extinction, reinstatemen and reacquistion. Since analysis of SCR-data only revealed group differences in fear expression during the re-extinction phase on day 2, we focused fMRI-analysis on this experimental phase.

During second-level analysis, independent t-test was used to examine group differences and one-sample t-tests were used to examine stimulus-specific group effects both for the sample as a whole and for each group separately. Given the dearth of previous studies on the effect of mindfulness meditation on extinction recall, we did not use previously defined regions of interest, but rather took an explorative approach focusing on whole-brain analysis. To correct for multiple comparisons, we used cluster-based correction, using a cluster-defining threshold of *p*<.01 uncorrected and a cluster level threshold of *p*<.05 FDR-corrected. All analyses were conducted using SPM12 (fil.ion.ucl.ac.uk/spm/software/spm12). Brain images in the figures were created using MRIcroGL (available at https://www.nitrc.org/projects/mricrogl/*).* For illustrative purposes, cluster edges are smoothed using the built-in smoothing tool in MRIcroGL.

## Results

### Self-report outcomes

**Fig. 2:**
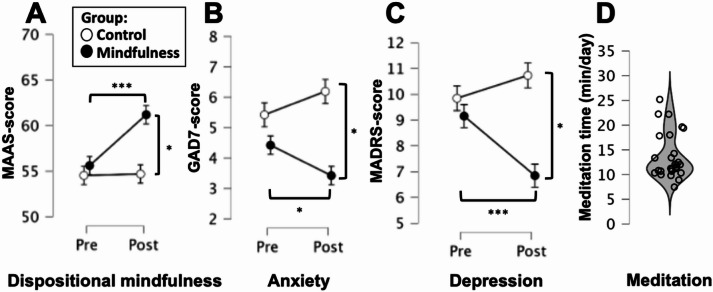
Self-report outcomes and intervention compliance. Dispositional mindfulness as well as anxiety and depression symptoms were measured before and after the intervention. For all three measures, there were no group differences before the intervention, but significant differences were observed after treatment. For all three measurements, the control group (*n* = 27) had similar scores pre- and post-intervention, whereas the mindfulness group (*n* = 26) showed increases in dispositional mindfulness (**A**), and decreases in anxiety (**B**) and depression symptoms (**C**). Additionally, compliance to the intervention was investigated with user-data extracted from the app, reflecting the amount of time participants spent doing guided meditation exercises. All participants in the mindfulness group (*n* = 27) showed acceptable compliance, where most spent an average of around 12 min/day meditating with the app (**D**).

There were no significant group differences before the intervention in terms of either dispositional mindfulness (MAAS-scores), anxiety (GAD-7) or depression (MADRS) symptoms (Fig. 2). However, group differences emerged after the intervention for all three measures, driven by changes in the mindfulness group with higher ratings for dispositional mindfulness and lower ratings for anxiety and depression for the mindfulness group relative to the waitlist control group.

To ascertain that there were no group differences in self-reported dispositional mindfulness before treatment, as well as to investigate intervention effects on this outcome, MAAS-scores were entered into a 2 × 2 rmANOVA with factors Time (pre; post) and Group (MF; WL).

Groups were highly similar in terms of dispositional mindfulness before the intervention, and mindfulness over four weeks was associated with robust increases in dispositional mindfulness, confirming an effect on this target outcome. rmANOVA showed a main effect of Time (F(1,51) = 8.48; *p*=.005) as well as a Time*Group interaction (F(1,51) = 7.63; *p*=.008, η²=0.023), but no main effect of Group (F(1,51) = 3.14; *p*=.083). Simple main effect analysis showed no main effect of group before treatment (F(1,51) = 0.24; *p*=.628) but a substantial effect after treatment (F(1,51) = 7.52; *p*=.008; η²=0.129), and that the mindfulness group showed a main effect of time (F(1,25) = 15.52; *p*<.001; η²=0.383) driven by increases in MAAS-scores, whereas the control group remained unchanged (F(1,26) = 0.01; *p*=.914), see Fig. 2A.

Similarly, we investigated self-reported levels of anxiety (GAD-7) and depression (MADRS) before the intervention, in order to confirm that the sample had low levels of psychopathological symptoms and did not differ before the intervention. No group differences were observed before the intervention either for GAD-7 (t(51) = 0.95; *p*=.344) or MADRS (t(51) = 0.51; *p*=.141), and descriptive data showed that for both measures, the sample as a whole scored in the higher end of the non-clinical range (GAD-7: M = 4.89; SD = 3.47; MADRS: M = 9.51; SD = 4.91).

Additionally, to investigate intervention effects on these outcomes, anxiety and depression scores were analyzed using 2 × 2 rmANOVAs with factors Time (pre; post) and Group (MF; WL). For anxiety we found a significant Time*Group interaction (F(1,51) = 6.91; *p*=.011; η²=0.018) and a main effect of Group (F(1,51) = 4.58; *p*=.037), but no main effect of Time F(1,51) = 0.07; *p*=.790). Simple main effect analysis showed a main effect of group after the intervention (F(1,51) = 9.61; *p*=.003; η²=0.159), but not before (F(1,51) = 0.91; *p*=.344), and that the mindfulness group showed a decrease in GAD-7 scores from pre- to post (F(1,25) = 5.51; *p*=.027; η²=0.181) whereas the control group had similar scores before and after the intervention (F(1,26) = 2.24; *p*=.143), see Fig. 2B. Similarly, for depression scores, we found a significant Time*Group interaction (F(1,51) = 11.36; *p*=.001; η²=0.021) but no main effect of Time (F(1,51) = 2.79; *p*=.101) or Group (F(1,51) = 2.66; *p*=.109). Again, simple main effect analysis showed a main effect of group after the intervention (F(1,51 = 6.01; *p*=.017; η²=0.107) but not before (F(1,51) = 0.26; *p*=.609), and that the mindfulness group decreased their MADRS-score pre- to post-intervention (F(1,25) = 13.44; *p*=.001; η²=0.350), whereas the control group did not show any changes (F(1,26) = 1.38; *p*=.251), see Fig. 2C.

### Intervention compliance

To verify adequate compliance with the intervention protocol, user-data from the app was extracted for the mindfulness group. This reflects the total amount of time that participants engaged in the guided mindfulness exercises using the Headspace app throughout the study period. All participants in the mindfulness group (*n* = 27) showed acceptable compliance, where the average usage time was 13.7 min/day (SD = 4.6 min/day; Min = 7.5 min/day; Max = 25.2 min/day). A majority of the sample were tightly clustered around 12 min/day (Median = 12.0 min/day), with a few participants (*n* = 7) exceeding 15 min/day, see Fig. 2D.

### SCRs: acquisition and extinction

**Fig. 3:**
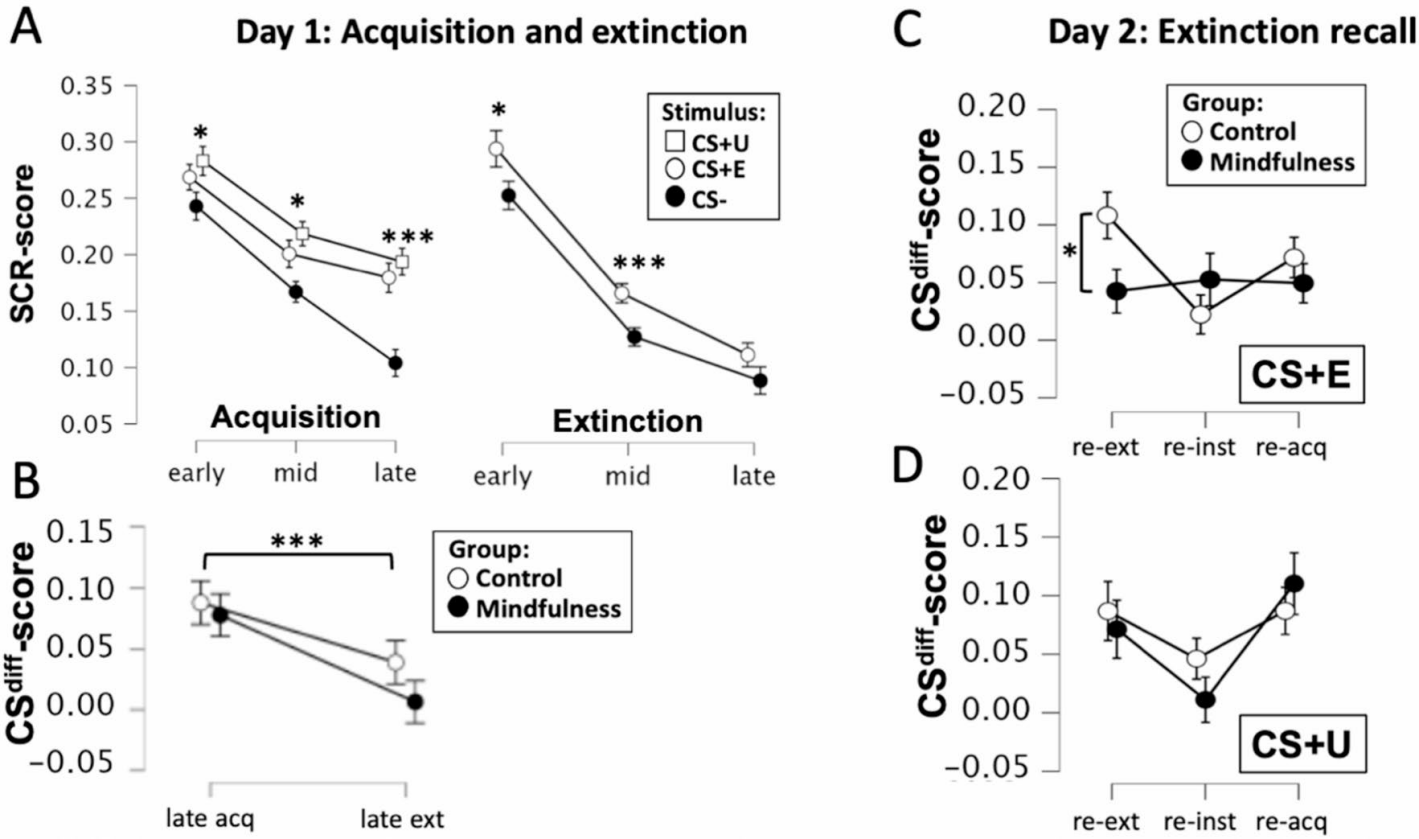
Results for skin conductance responses days 1 and 2. (**A**). During acquisition participants (*n* = 54) showed stronger SCRs to the CS + E and CS + U compared to the CS-, with larger differences for later trials, whereas CS + E and CS + U responses were highly similar, and SCRs did not differ across groups (MF = 27; WL = 27). During extinction, overall, CS+ responses were stronger than CS- responses, but this difference was substantially reduced in later trials, again with no group differences. Plots show means and SEMs for root-transformed and range-corrected SCRs, both groups combined. Asterisks indicate whether a main effect of stimulus was present for each phase (*p<.05; ***p<.001). (**B**). In order to verify that extinction was achieved, we calculated a CS-differentiation index (CS^diff^), by subtracting CS- responses from CS+ responses, for late acquisition and extinction respectively, to directly test for decreases in CS differentiation from end of acquisition to end of extinction. Results indicated successful extinction in both groups equally (MF = 27; WL = 27), with substantial decreases in CS^diff^-scores (*p*<.001), and no between groups effects. (**C**) and (**D**). To evaluate extinction-recall, CS^diff^-scores for the CS + E and CS + U were calculated, and compared across groups and phases. The results showed a three-way interaction between phase, CS-type and group (*p*=.026), where a group difference (*p*=.028) was observed only for the extinguished CS+, and only during early extinction recall, i.e. the re-extinction phase (**C**). For this phase specifically, the mindfulness group (*n* = 27) showed lower CS^diff^-scores compared to the control group (*n* = 28), whereas groups showed similar responses to the non-extinguished CS+ during all phases (**D**), supporting the conclusion that the mindfulness-intervention had a positive effect on extinction recall. Plots display means and SEM.

To examine effects of fear acquisition, event-related SCRs for non-reinforced stimulus presentations were entered into a 3 × 3 × 2 rm-ANOVA with factors Stimulus (CS + E; CS + U; CS-), Time (Early; Mid; Late) and Group (MF; WL). Results showed a main effect of Stimulus (F(2,104) = 18.57; *p*<.001), with stronger responses to both reinforced cues as compared to the CS-, a main effect of Time (F(2,104) = 38.78; *p*<.001) with diminishing responses with time, and a Stimulus*Time interaction (F(4,208) = 2.87; *p*=.024) reflecting larger effects of Stimulus for late time-points, but no other main effects or interactions (*p*>.095). Simple main effects analysis with Stimulus as the simple effect factor and Time as the moderator factor showed main effects of Stimulus across all time points (early: F(2,104) = 4.75; *p*=.011; mid: F(2,104) = 6.89; *p*=.002; late: F(2,104) = 17.18; *p*<.001), but with the strongest effect during late acquisition, see Fig. 3A. Taken together, this indicates that the acquisition was successful, resulting in differential responses to CS+ compared to CS-, with no group differences. To ascertain that responses did not differ for CS + E and CS + NE during acquisition we similarly performed a 2 × 3 × 2 rm-ANOVA with factors Stimulus (CS + E; CS + NE), Time (Early; Mid; Late) and Group (MF; WL). As expected, the results showed a main effect of Time (F(2,104) = 22.62; *p*<.001), with diminishing responses across trials, but no other main effects or interactions (> 0.081).

To further verify that differential CS responding was achieved by the end of acquisition, and did not differ between groups, we performed two separate 2 × 2 mixed ANOVAs, with factors Stimulus (CS + E/CS + U; CS-) and Group (MF; WL), for the late acquisition phase for both the CS + E and CS + U respectively. For both the CS + E vs. CS- and CS + U vs. CS-, we found a main effect of stimulus (CS + E: F(1,52) = 26.04; *p*<.001; CS + U: F(1,52) = 28.70; *p*<.001), but no other main effects or interactions (*p*<.153). Thus, we observed successful separation vs. CS- for both CS + E and CS + U by late acquisition, with no group differences.

To examine effects of extinction, SCRs from the extinction phase were entered into a 2 × 3 × 2 rm-ANOVA with factors Stimulus (CS + E; CS-), Time (early, mid, late) and Group (MF; WL). Results showed a main effect of Stimulus (F(1,52) = 13.21; *p*<.001) with larger responses to the CS + E compared to the CS-, and a main effect of Time (F(2,104) = 86.08; *p*<.001) with diminishing responses for later time points, but no other main effects or interactions (all ps>0.268). Notably, simple main effects analysis with Stimulus as the simple effect factor and Time as the moderator factor showed CS differentiation during early (F(1,52) = 5.38; *p*=.024) and mid-extinction (F(1,52) = 11.38; *p*=.001) but not during late extinction (F(1,52) = 3.20; *p*=.079), see Fig. 3A. As a more direct test of extinction effects, we next evaluated whether CS differentiation decreased from the end of acquisition to the end of extinction. Thus, we calculated CS difference scores (CS^diff^) by subtracting CS- responses from the CS+ responses for the late acquisition and extinction phase respectively, and entered these values into a 2 × 2 rm-ANOVA with factors Phase (late acquisition; late extinction) and Group (MF; WL). The results showed a main effect of Phase (F(1,52) = 11.64; *p*=.001), where CS^diff^-scores decreased from late acquisition to late extinction, but no main effect of Group (F(1,52) = 1.29; *p*=.261), and no Phase*Group interaction (F(1,52) = 0.39; *p*=.531). Furthermore, simple main effect analysis investigating the main effect of Group for each phase respectively, showed no group differences either for late acquisition (F(1,52 = 0.15; *p*=.699) or late extinction (F(1,52 = 1.65; *p*=.205). Taken together these analyses show that successful extinction was achieved, in which CS differentiation decreased substantially from late acquisition to late extinction. Furthermore, this decrease was of similar magnitude in both groups, and that groups did not differ in CS differentiation during either phase, thus demonstrating comparable levels of acquisition and extinction of conditioned threat responses.

### SCRs: extinction recall

The main research question remains whether there are intervention effects of mindfulness training on extinction recall. To evaluate this differential responding to CS + E and CS + U vs. CS- during the recall test on day 2 was evaluated. For this analysis, we extracted CS^diff^-scores by subtracting CS- responses from CS + E and CS + U responses respectively and calculated average CS+E^diff^ and CS+U^diff^-scores for each phase (re-extinction, reinstatement and reacquisition). These were then entered into a 3 × 2 × 2 rm-ANOVA with factors Phase (re-ext; re-inst; re-acq), Stimulus (CS+E^diff^; CS+U^diff)^ and Group (MF; WL). The results showed a main effect of Phase (F(2,106) = 4.10; *p*=.019) and a Phase*Stimulus*Group interaction (F(2,106) = 4.00; *p*=.026; η²=0.011), but no other main effects or interactions (all ps>0.124). Pertaining to the main effect of Phase, post-hoc t-test showed that differential CS responses were larger during re-extinction (t(54) = 2.21; *p*=.031) and re-acquisition (t(54) = 3.05; *p*=.004), as compared to reinstatement, suggesting that the unsignaled shocks presented at the beginning of the re-instatement phase did not have the intended effect of potentiating differential CS responding. Rather, the result indicates that differential CS responding was still intact during re-extinction, indicating spontaneous recovery of threat responses. In other words, threat responses were diminished with additional non-reinforced trials during reinstatement, but then increased with additional reinforced trials during reacquisition. In terms of the Phase*Stimulus*Group interactions, simple main effects analysis with Group as the simple effect factor, and Phase and Stimulus as moderator factors, showed group differences only for the CS + E during the re-extinction phase with a medium effect-size (F(1,53) = 5.10; *p*=.028; η²=0.088), and no group differences for either CS during any of the other phases (all ps>0.252). These results indicate that the mindfulness group had diminished threat responses specifically to the extinguished CS+ during the first phase of the extinction recall test procedure. Taken together, these results suggest that mindfulness improves extinction recall, as seen through diminished threat-responses during early re-extinction, in line with our previous observations^8^. Additionally, these findings show that this effect is specific to cues that have undergone extinction, supporting the conclusion that mindfulness specifically impacts the extinction processes, rather than conditioned threat responses as a whole.

### fMRI

**Fig. 4:**
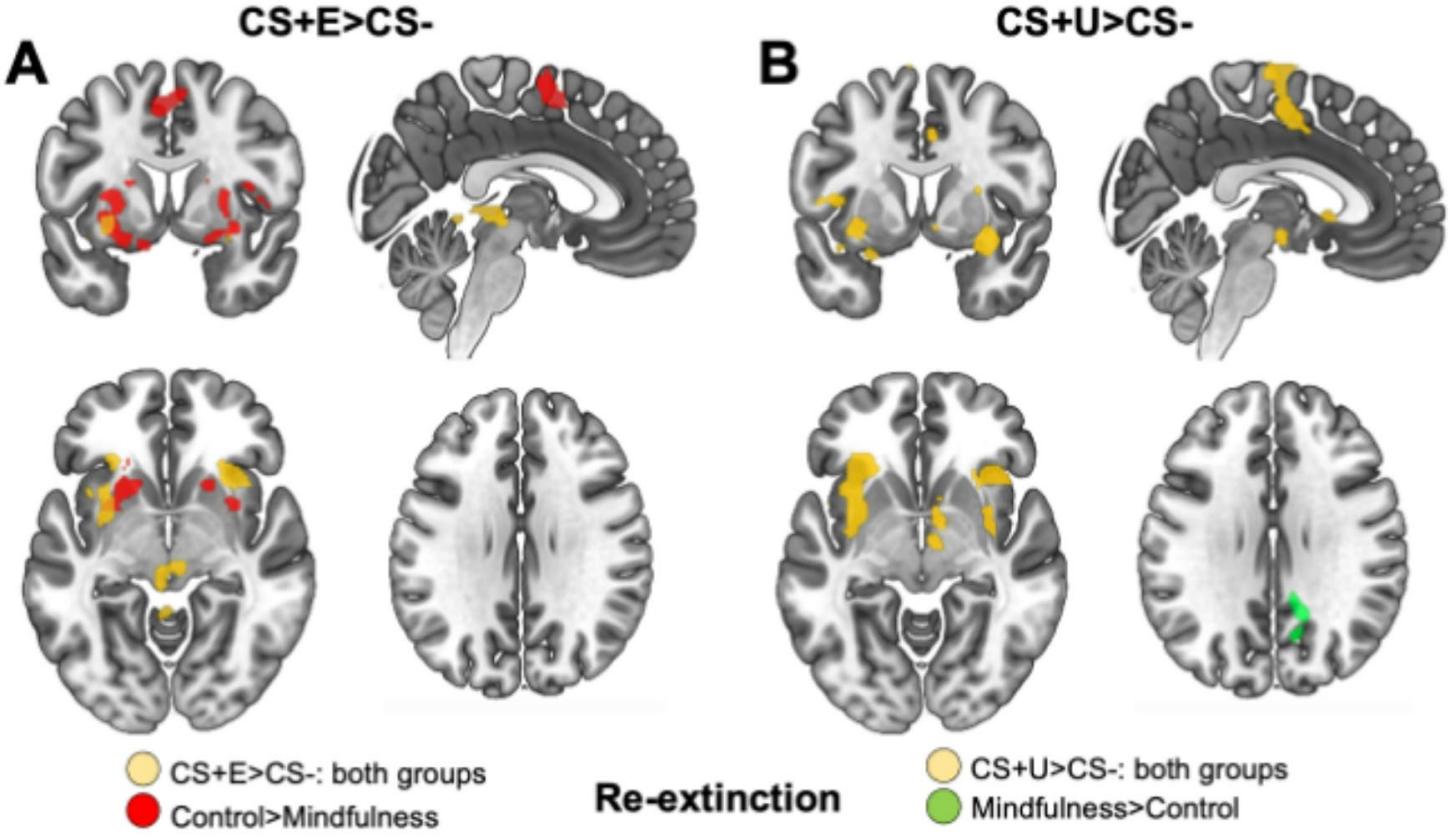
Activation patterns and group differences during early extinction recall for the extinguished (CS + E) and non-extinguished (CS + U) threat-cue. (**A**). Activations for CS + E vs. CS-: During the re-extinction phase on day 2 we found CS + E> CS- activations across both groups (MF *n* = 25; WL *n* = 24) in several regions consistently associated with threat processing, including bilateral anterior insula, lateral putamen, and midbrain regions including the PAG (yellow colors). Additionally, we found stronger activations for the control group in several threat-related regions. Between-group effects were observed in three clusters including bilateral striatum (primarily lateral putamen and ventral striatum) extending into dorsal amygdala, and one cluster in the SMA (red colors). (**B**). Activations for CS + U vs. CS-: For comparison, we performed the same analysis for the non-extinguished cue. Across both groups we found extensive CS + U> CS- activation in several regions related to threat-processing, including two bilateral clusters encompassing anterior insula, extending into operculum, lateral putamen and dorsal amygdala, and a medial frontal cluster encompassing mid-cingulum, SMA and pre-central gyrus (yellow colors). Additionally, we found stronger activation in the mindfulness group in one cluster encompassing right precuneus and adjacent white-matter tracts (green colors), although this region is not implicated in threat processing in previous studies. Images show only voxels passing the corrected threshold, projected on a standardized brain template (MNI152) using neurological convention (left is left).

Since analysis of SCRs indicated that group differences in threat responses were only present during re-extinction, we focused brain-imaging analysis on this experimental phase. To this end, we investigated effects for the CS + E vs. CS- contrast during re-extinction, and for comparison, performed similar analyses for the CS + U vs. CS- contrast. First, to investigate common activations across both groups, we analyzed the CS + E> CS- contrast across all participants. Here, we found activations in two bilateral clusters encompassing the left and right anterior insula extending in to the lateral putamen (left: *Z* = 4.25, *P*_FDR_ = 0.005, 3360 mm^3^, MNI peak voxel: -32, 2, -8; right: *Z* = 4.80, *P*_FDR_ = 0.005, 3504 mm^3^, MNI peak voxel: 28, 20, -4), as well as a midbrain cluster encompassing the periaqueductal gray (PAG) extending into dorsal cerebellum (*Z* = 4.56, *P*_FDR_ = 0.040, 2144 mm^3^, MNI peak voxel: −22, 0, − 14), see Fig. 3A. For the CS-> CS + E contrast, we found one cluster encompassing the right precentral gyrus (*Z* = 3.91, *P*_FDR_ = 0.006, 3552 mm^3^, MNI peak voxel: 2, -38, 72). Both the locations and direction of effects in these clusters are consistent with previous large-scale studies on fear conditioning and extinction, as regions that are typically activated/de-activated in CS + vs. CS- comparisons^70,32,71^. Then, to evaluate the main research question, we looked at group differences for the CS + E vs. CS- contrast. For the MF > WL comparison we did not find any clusters, but for the WL > MF comparison three clusters were observed indicating larger activity in the control group compared to the mindfulness group, see Figs. 3A and 4. One cluster in the supplemental motor area (*Z* = 3.36, *P*_FDR_ = 0.023, 2320 mm^3^, MNI peak voxel: 4, 2, 56), and two bilateral clusters mainly encompassing lateral putamen and ventral striatum, extending into dorsal caudate and dorsal amygdala (left: *Z* = 4.12, *P*_FDR_ <0.001, 5888 mm^3^, MNI peak voxel: -18, 2, -12; right: *Z* = 3.27, *P*_FDR_ = 0.003, 3560 mm^3^, MNI peak voxel: 30, 0, -10). These are also regions that are typically activated for CS + > CS- contrasts in large scale neuroimaging studies^32,72^, and are considered part of the fear network, specifically engaged during threat processing.

Notably, these group differences could be due either to increased activity in the control group, activity decreases in the mindfulness group, or a combination. To investigate this, we performed exploratory in stimulus-specific group analyses, looking at both the CS + E> CS- as well as CS-> CS + E contrast in each group separately, using a functional ROI corresponding to the regions where group differences were observed using a more lenient threshold (*p*>.01, uncorrected; minimal cluster extent of 20 voxels). For the control group, the CS + E> CS- contrast showed substantial activation in almost all parts of the identified regions, consisting of four observed clusters, two clusters in the left and right ventral striatum and lateral putamen, extending into dorsal amygdala (left: *Z* = 4.88, *P*_uncorr_<0.001, 3040 mm^3^, MNI peak voxel: -32, 2, -8; right: *Z* = 3.91, *P*_uncorr_ < 0.001, 2600 mm^3^, MNI peak voxel: 22, 2, -12), one cluster in the SMA (*Z* = 3.67, *P*_uncorr_ < 0.001, 1320 mm^3^, MNI peak voxel: -6, 4, 54) and one cluster in left caudate (*Z* = 3.24, *P*_uncorr_ = 0.001, 200 mm^3^, MNI peak voxel: -12, -2, 12), whereas no effects were observed for the CS-> CS + E contrast. For the mindfulness group no clusters were observed for the CS + E > CS- contrast, but two clusters were found for the CS-> CS + E contrast, located in left ventral striatum (*Z* = 3.03, *P*_uncorr_ = 0.001, 240 mm^3^, MNI peak voxel: -20, 12, -6) and left dorsal caudate (*Z* = 3.72, *P*_uncorr_ < 0.001, 520 mm^3^, MNI peak voxel: -18, -2, 20), see Fig. 4.

Furthermore, to examine whether these effects were specific to the extinguished CS+, we performed the same analysis for the CS + U vs. CS- contrast for comparison. First, to investigate common activations across both groups, we analyzed the CS + U vs. CS- contrast across all participants. Here, we found activations in four clusters encompassing large parts of the fear network. One medial cluster extending from the midcingulate region into the SMA, and precentral gyrus (*Z* = 4.65, *P*_FDR_ < 0.001, 12 520 mm^3^, MNI peak voxel: -10, -14, 68), two lateral clusters encompassing the anterior and middle insula, extending into operculum, lateral putamen and dorsal amygdala (left: *Z* = 4.21, *P*_FDR_ < 0.001, 9 376 mm^3^, MNI peak voxel: -62, 24, -10; right: *Z* = 4.40, *P*_FDR_ < 0.001, 8 016 mm^3^, MNI peak voxel: 30, 2, -14), and one smaller medial subcortical cluster primarily on the right side encompassing ventral striatum, pallidum, hypothalamic regions, thalamus, anterior midbrain and adjacent white matter tracts (*Z* = 3.68, *P*_FDR_ = 0.024, 2 240 mm^3^, MNI peak voxel: 8, 0, -6), see Fig. 4B. For the CS-> CS + U contrast, we found two clusters, one in the right dlPFC (*Z* = 3.40, *P*_FDR_ = 0.036, 2 264 mm^3^, MNI peak voxel: −24, 22, 58) and one in the superior division of the right lateral occipital cortex, (*Z* = 3.60, *P*_FDR_ = 0.030, 2 720 mm^3^, MNI peak voxel: 32, -82, 42). Secondly, looking at group comparisons, for the WL > MF contrast no clusters were observed, and for the MF > WL contrast we found one cluster largely encompassing white matter, but also extending into right precuneus and posterior cingulate gyrus (*Z* = 3.58, *P*_FDR_ = 0.033, 2 784 mm^3^, MNI peak voxel: 16, -52, 38), see Fig. 3B. Taken together, these results are consistent with the effects that were observed for SCR-data. For the CS + E vs. CS- comparison, activations of several nodes of the fear network were observed across both groups, but group differences were also observed in several regions (i.e. SMA, striatum and dorsal amygdala) with stronger activations in the control group. Notably, group differences were observed in areas associated with threat processing (i.e. consistent with CS + > CS- activity patterns) rather than regions associated with fear inhibition (i.e. consistent with CS-> CS+ activity patterns). Whereas the CS + U vs. CS- comparison showed robust activation of several regions related to threat processing across both groups, no clear group differences in activation patterns in regions related to threat processing emerged. Overall, this supports the conclusion that the mindfulness intervention improved extinction recall, as shown for both threat-related physiological arousal and neural processing, specifically for extinguished threat-cues.

**Fig. 5:**
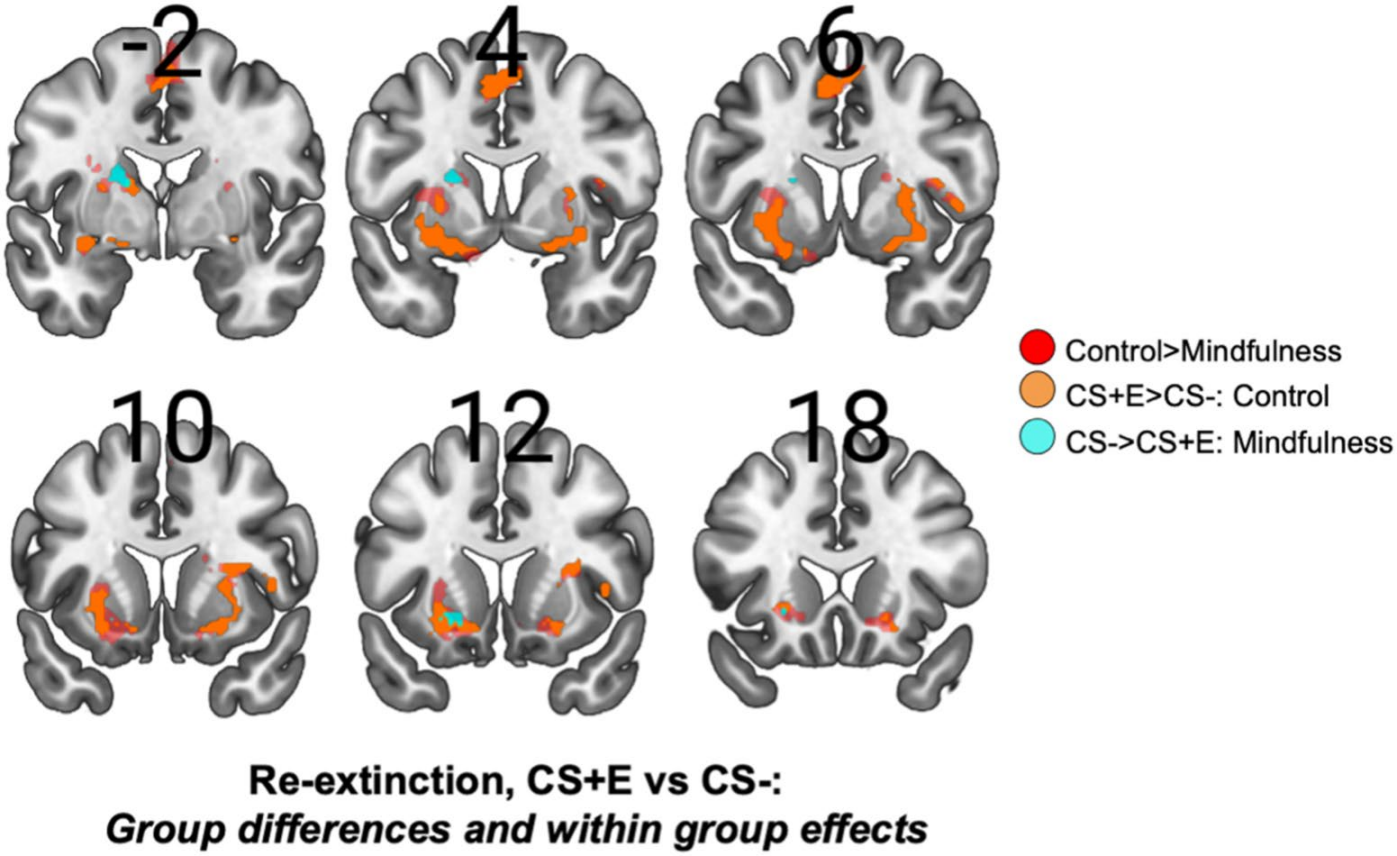
Group activation patterns during early extinction recall for the extinguished threat-cue (CS + E). Group differences observed for the CS + E> CS- comparison, where the control group showed higher activity, could be driven both by activity increases in controls or decreases in the mindfulness group. To clarify this, we investigated activations and deactivation for CS + E vs. CS- contrast within each group separately, specifically looking at areas where group differences were observed (red colors). The controls showed extensive activations in most parts of this ROI (orange colors) and no deactivations. Contrary to this, the mindfulness group showed no activations, but rather deactivations in two parts of the left striatum (turquoise colors), indicating that between group-effects are largely driven by increased activity in the controls. Images show voxels passing an uncorrected threshold (*p*<.01, minimum of 20 voxels), projected on a standardized brain template.

### Brain-behavior correlations

To further explore the association between neural activation and physiological threat-responses during extinction recall we carried out analysis of possible brain-behavior correlations. For the CS + E vs. CS- contrast, we added the CS+E^diff^-score as a regressor to the model and investigated positive and negative associations between threat related SCRs and BOLD-activations, and similarly performed between-group analysis for these associations. Firstly, we examined possible brain-behavior correlations in the regions where we observed group differences, adding these clusters as the ROI. Using a corrected threshold, no positive or negative associations were observed either for the sample as whole, or in any of the groups separately. However, exploratory analysis applying an uncorrected threshold (*p*<.01, minimum of 20 voxels) revealed a positive association in a small cluster in the control group located in the transition zone between left dorsal amygdala and left ventral striatum (*Z* = 3.47, *P*_uncorr_ < 0.001, 176 mm^3^, MNI peak voxel: -18, 2, -16), see Fig. 5, left panel, but no other positive or negative association in either group, nor any group differences. Correspondingly, correlation analysis using extracted beta-values for this cluster showed a strong positive association in the control group (*r* = .61; *p* = .001), but no clear association in the mindfulness group (*r* = − .25 ; *p* = .225), see Fig. 5, right panel.

**Fig. 6:**
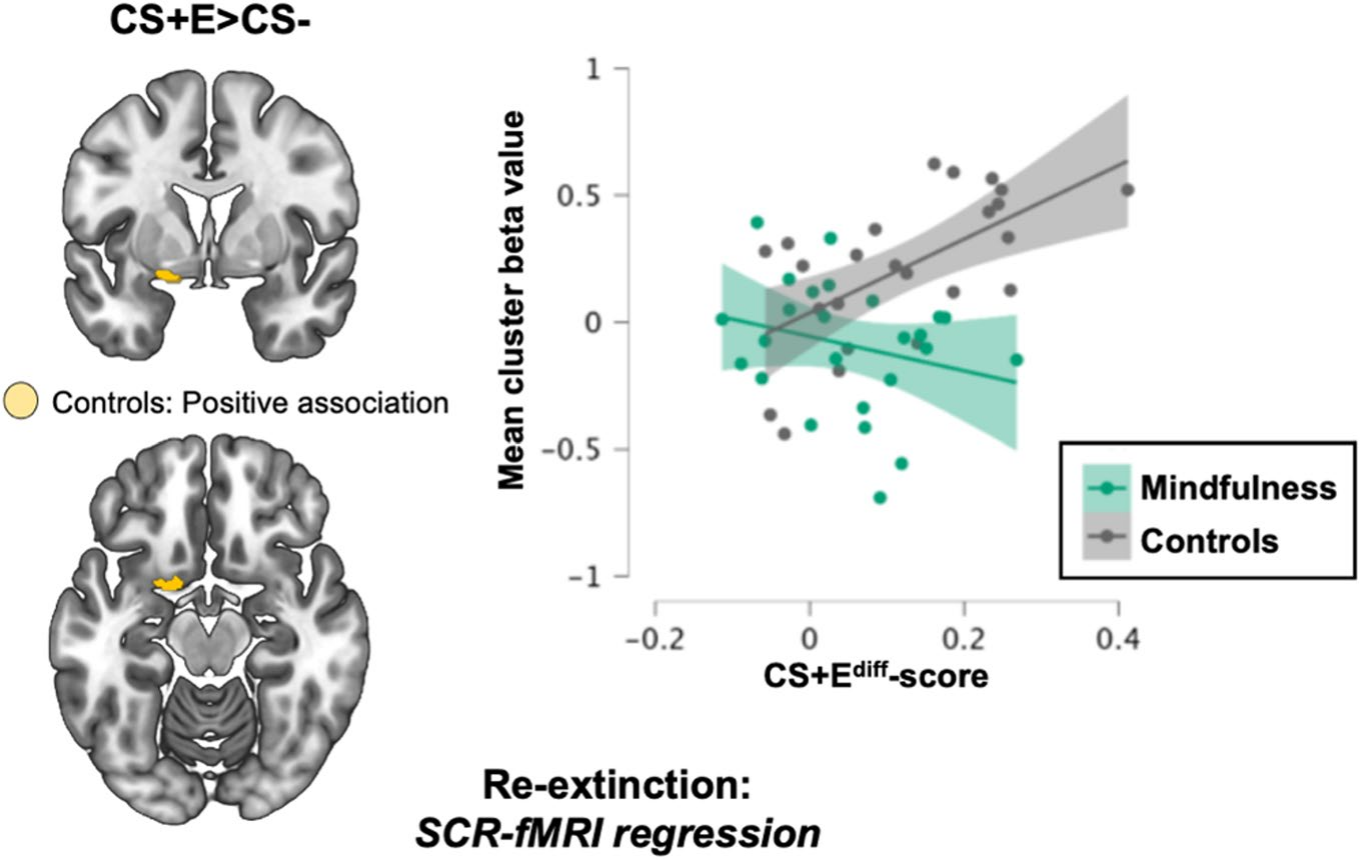
Association between skin conductance and neural activation in regions with between-group effects. Exploratory analysis using an uncorrected threshold (*p*<.01, minimum of 20 voxels), and an ROI corresponding to the three clusters where group differences were observed (see Fig. 4) showed a positive correlation between CS+E^diff^-scores and fMRI-BOLD for the CS + E vs. CS- contrast for controls (*n* = 24), but not the mindfulness group (*n* = 25), in a small region in the transition zone between dorsal amygdala and ventral striatum, on the left side. No associations were observed using corrected thresholds. Left panel shows the location of this cluster projected on a standardized brain template. Right panel shows the association for each group separately with regression lines and 95%-confidence intervals.

**Fig. 7:**
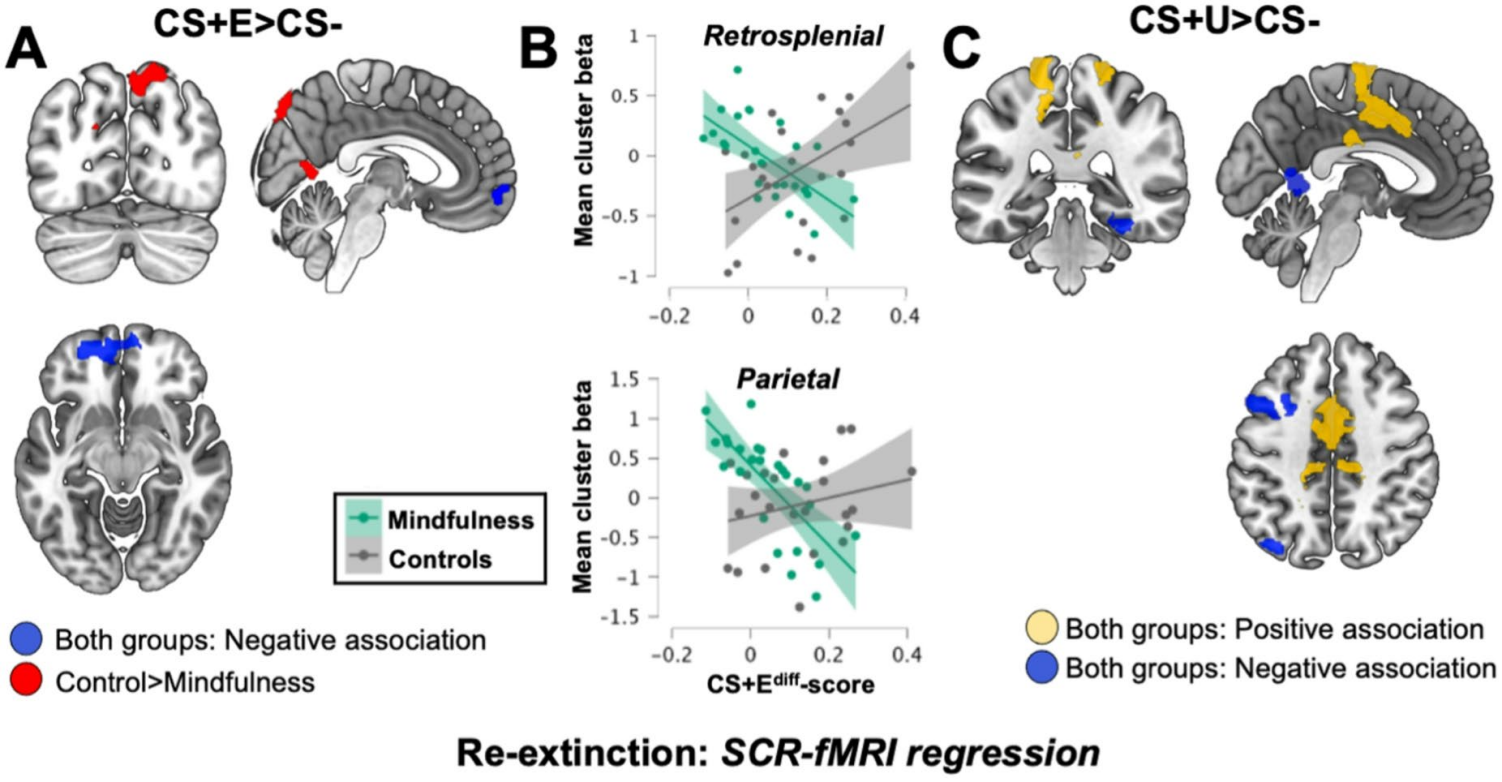
Association between skin conductance and neural activation during early extinction recall for the extinguished (CS + E) and non-extinguished (CS + U) threat-cue, whole-brain results. (**A**). For both groups combined we found a negative correlation between CS+E^diff^-scores and fMRI-BOLD for the CS + E vs. CS- contrast, in one cluster in the anterior vmPFC (blue colors). Exploratory analysis using liberal thresholds did not reveal any group differences for this association. The negative correlation was strong and very similar in both the mindfulness group and controls. Additionally, we found group differences for SCR-fMRI association (Controls> Mindfulness), in two clusters located in the right posterior parietal cortex and retrosplenial region (red colors). (**B**). Stimulus-specific group correlations using extracted beta-values from these clusters showed that for the posterior parietal cluster, group differences are driven by a negative association in the mindfulness group (bottom panel), and for the retrosplenial cluster a combination of negative correlation in the mindfulness group and a positive correlation in controls (top panel). Plots show the association for each group separately with regression lines and 95%-confidence intervals. (**C**). For comparison we similarly investigated SCR-fMRI correlations for the CS + U vs. CS- comparison. For both groups combined we found both positive (yellow colors) and negative (blue colors) associations in several regions implicated in threat-processing in previous studies, but no group differences. Images show only voxels passing the corrected threshold, projected on a standardized brain template.

Secondly, we repeated this analysis looking at whole-brain results using corrected thresholds. In the sample as a whole, no clusters were observed for positive associations. However, for negative associations one cluster in the anterior vmPFC was identified (*Z* = 4.38, *P*_FDR_ = 0.002, 4280 mm^3^, MNI peak voxel: -24, 52, -12), see Fig. 6A. Voxel-wise analysis revealed no group differences for this cluster, either using a corrected threshold or an uncorrected threshold (*p*<.05 uncorrected, minimal cluster extent of 20 voxels). Furthermore, using average extracted beta-values from the entire cluster and performing Pearson correlation showed that the association was strong (*r* = -.52; *p*<.001) and highly similar in both groups (WL: *r* = -.61; *p* = .002; MF: *r* = .59; *p* = .002). This suggests that the vmPFC has an inhibitory influence over fear expression when viewing an extinguished CS+ during extinction recall, but this effect does not appear to be influenced by mindfulness. Looking at group differences, for the MF > WL contrast, no clusters were observed, but for the WL > MF contrast we found two clusters, one in the right medial posterior parietal lobe mainly encompassing precuneus (*Z* = 3.73, *P*_FDR_ = 0.025, 2760 mm^3^, MNI peak voxel: 16, -74, 56), and one medial cluster in the retrosplenial region (*Z* = 3.98, *P*_FDR_ = 0.025, 2512 mm^3^, MNI peak voxel: 8, 0, -6), see Fig. 6A. These group differences in brain-behavior associations could be due both to negative associations in the mindfulness group, positive associations in the control group, or a combination. In order to investigate this, we extracted average beta-values from these two clusters and performed group analyses, evaluating the correlation with CS+E^diff^-scores for each group separately. The results showed that the posterior parietal cluster primarily driven by a negative association in the mindfulness-group where we observed a strong negative correlation (*r* = -.72; *p*<.001). In contrast, no significant correlation was found for the WL-group (*r* = .26; *p*=.213) see Fig. 6B, bottom-panel. As for the retrosplenial cluster, this appears to be driven both by a negative association in the mindfulness group (*r* = -.62; *p*<.001), as well as a positive association in the control group (*r* = .49; *p* = .015), see Fig. 6B, top-panel Fig. 7).

For comparison, we similarly investigated brain-behavior correlations for the non-extinguished CS+, by adding CS+U^diff^-scores as a regressor to the CS + U vs. CS- contrast. Here we found no group differences, but looking across both groups we observed positive association between CS+U^diff^-scores and fMRI-BOLD-responses in 4 clusters, see Fig. 6C. Two parietal clusters located in left (*Z* = 4.24, *P*_FDR_ < 0.001, 7856 mm^3^, MNI peak voxel: -14, -44, 78) and right (*Z* = 3.48, *P*_FDR_ = 0.008, 2752 mm^3^, MNI peak voxel: 20, -50, 68) postcentral gyrus (PCG), where the left side cluster was more extensive, extending ventrally into the medial PCG, one medial frontal cluster extending from SMA down into dACC (*Z* = 4.12, *P*_FDR_ < 0.001, 12 328 mm^3^, MNI peak voxel: 2, -8, 74), and one cluster in the mid-cingulate region (*Z* = 4.04, *P*_FDR_ = 0.001, 4120 mm^3^, MNI peak voxel: 8, 0, -6). All of these clusters largely overlap with regions related to threat processing in previous studies^32,70,73^. Looking at negative associations, across both groups we found six clusters encompassing the following regions; one cluster left dlPFC encompassing middle and superior frontal gyrus (*Z* = 4.43, *P*_FDR_ < 0.001, 11 568 mm^3^, MNI peak voxel: -28, 18, 60), one cluster mostly encompassing the superior division of the left lateral occipital cortex (*Z* = 3.83, *P*_FDR_ = 0.001, 4424 mm^3^, MNI peak voxel: -30, -72, 48), one cluster in the retrosplenial region (*Z* = 4.32, *P*_FDR_ = 0.002, 3696 mm^3^, MNI peak voxel: 4, -50, 2), one cluster in the left posterior medial temporal lobe mostly encompassing parahippocampal gyrus and the temporal fusiform, extending into hippocampus (*Z* = 3.98, *P*_FDR_ = 0.003, 3416 mm^3^, MNI peak voxel: 26, -20, -30), one cluster in left orbitofrontal cortex (*Z* = 3.55, *P*_FDR_ = 0.021, 2224 mm^3^, MNI peak voxel: -38, 38, 6), and one cluster in the right inferior temporal gyrus (*Z* = 3.62, *P*_FDR_ = 0.028, 2000 mm^3^, MNI peak voxel: 50, -72, 16). Overall, the location of these clusters is fairly consistent with activation patterns that are observed for CS-> CS+ contrasts (i.e. deactivations) in previous imaging studies^32,72^, supporting the conclusion that they may be related to inhibition of conditioned threat responses.

## Discussion

Here we examined the neural and physiological effects of a 30-day mindfulness intervention on conditioned threat responses, specifically extinction recall, to explore how mindfulness may mitigate fear responses. In a healthy cohort randomly allocated to either a mindfulness group or waitlist control group with no or minimal previous experience of mindfulness, we looked at both physiological threat responses (SCRs) and neural activity patterns (fMRI). In line with our previous findings^8^, we found that participants in the mindfulness-group showed lower threat responses during early extinction recall compared to a non-intervention control group. Furthermore, this effect was specific to the extinguished threat-cue, supporting the conclusion that mindfulness has a specific effect on extinction related processes, rather than a general effect on delayed threat responding. Analysis of threat responses during fear acquisition and extinction on day 1 indicated that SCRs were robustly increased to conditioned cues during acquisition, and then diminished during extinction, to a similar degree in both groups, with group differences only evident for the recall-test on day 2, suggesting that the mindfulness intervention may uniquely impact extinction recall. The mindfulness group showed diminished activation in key fear response areas, such as the striatum and amygdala, as well as the SMA, only for the extinguished CS+ stimuli. In contrast, the group as a whole showed activations of anterior insula and PAG, suggesting that only certain parts of the fear-network are affected by mindfulness. Taken together, pattern is consistent with the interpretation that mindfulness interacts specifically with extinction learning of fear, rather than reducing global fear responses. However, it should be noted that the absence of pre-intervention fear conditioning measures means that we cannot rule out the possibility that pre-existing group differences in extinction recall capacity, despite randomization, may have contributed to the observed effects. Notably, we did not observe evidence that mindfulness enhanced extinction recall through a top-down inhibitory mechanism involving increased vmPFC or hippocampal activity, although we cannot rule out that our study may have been underpowered to detect such effects. We observed that vmPFC activity was negatively correlated with threat responses to extinguished cues across both groups, consistent with its established role in extinction^30^, this relationship was not enhanced by mindfulness training. Instead, our results are consistent with a modulation of subcortical threat-processing regions, suggesting a possible alternative mechanism by which mindfulness influences fear extinction. The observed reductions in amygdala, striatum, and SMA activation specifically to extinguished threat cues are consistent with the possibility that mindfulness training may alter the responsivity of threat-processing circuits, although we note that the locus of observed neural change does not necessarily indicate the source of that change, and top-down mechanisms may still contribute. This pattern aligns with evidence that mindfulness practice leads to structural and functional changes in these regions^7,74^, but extends previous findings by demonstrating that these changes translate into specific improvements in safety memory retrieval. The absence of enhanced vmPFC or hippocampal activity in our mindfulness group contrasts with some theoretical predictions and previous findings^18,36^. However, this discrepancy may reflect important differences in study design and the specific mindfulness protocols employed. While Sevinc et al.,^36^, who had a larger neuroimaging sample (42 MBSR and 25 controls, though SCR analyses were limited to 8 per group due to methodological quality control), emphasized hippocampal contributions to extinction enhancement, our findings suggest a complementary mechanism that may involve subcortical modulation, potentially alongside top-down control processes.

We previously reported that mindfulness may improve extinction retention. These effects were specific to extinguished CS indicating that mindfulness impacts extinction processes specifically. The mindfulness group’s diminished activation of bilateral ventral striatum specifically for extinguished CS+ represents a novel finding that bridges fear extinction and reward processing literatures. The striatum plays a crucial role in prediction error signaling and salience attribution^75^, processes that are fundamental to both fear conditioning and extinction learning. Our findings suggest that mindfulness training may enhance extinction recall by reducing the salience attributed to previously threatening cues that have been designated as safe. This interpretation is supported by previous work showing that emotion regulation strategies reduce both physiological responses and ventral striatal activity to emotional stimuli^76^, and that mindfulness training attenuates prediction error signals in striatal regions^77^. The specific reduction in striatal responsivity to extinguished (but not non-extinguished) threat cues indicates that mindfulness enhances the precision of safety memory retrieval, allowing for more accurate discrimination between current threat and safety contexts.

Understanding the neural mechanisms by which mindfulness enhances extinction recall has direct implications for optimizing exposure-based treatments for anxiety and trauma-related disorders. Our findings suggest that mindfulness training could serve as an effective adjunct to cognitive-behavioral therapy by enhancing the consolidation and retrieval of safety memories formed during exposure sessions. The specific modulation of subcortical threat-processing circuits indicates that mindfulness may be particularly beneficial for patients who struggle with the emotional aspects of exposure therapy.

Furthermore, the implicit nature of these effects suggests that mindfulness-enhanced extinction recall may be less dependent on cognitive resources and conscious effort compared to traditional cognitive reappraisal strategies. This could make mindfulness-based interventions particularly valuable for populations with limited cognitive control capacity or high emotional reactivity. Future clinical trials should investigate whether pre-treatment mindfulness training enhances the effectiveness of exposure-based therapies.

Our findings are consistent with the hypothesis that mindfulness may influence extinction processes through implicit rather than explicit emotion regulation mechanisms. The specificity of effects to extinguished cues, combined with the absence of enhanced prefrontal control regions, is consistent with the possibility that participants were not consciously applying emotion regulation strategies during the task. However, as noted above, the observed locus of neural change in subcortical regions does not necessarily imply a bottom-up source of that change, and we cannot definitively exclude contributions from top-down mechanisms. The pattern of results is nonetheless consistent with the possibility that mindfulness training may produce changes in the responsivity of threat-processing circuits that could persist without requiring active deployment of conscious regulatory strategies during threat encounters.

### Limitations

Several limitations should be considered when interpreting these findings. First, the extinction recall phase included only four trials per condition, which may provide a limited estimate of extinction recall at the individual-subject level, and the relatively small sample size (*n* = 27 per group for SCR; *n* = 49 for fMRI) may limit statistical power for detecting training-related neural effects. These factors, combined with our use of cluster-wise inference with a cluster-defining threshold of *p* < .01 uncorrected, which can be considered liberal^78^, suggest that our findings should be considered preliminary and require replication with larger samples and more robust extinction recall measurement. Second, our study examined healthy participants with minimal prior mindfulness experience, and the generalizability to clinical populations remains to be established. Third, while our app-based intervention effectively enhanced extinction recall, the specific components of mindfulness training responsible for these effects (e.g., focused attention, present-moment awareness, acceptance) remain unclear. Fourth, our design did not include measures of emotion regulation strategies, limiting our ability to definitively rule out explicit regulatory mechanisms. Future research should examine dose-response relationships, investigate the optimal timing of mindfulness training relative to exposure therapy, and test these mechanisms in clinical populations with anxiety and trauma-related disorders. Additionally, studies incorporating real-time measures of attention and acceptance during fear conditioning could help elucidate the specific mindfulness components driving extinction enhancement. Fifth, our study did not include pre-intervention fear conditioning measures, which limits the ability to make strong causal claims about mindfulness-induced changes in extinction recall. While randomization should equalize baseline differences, future studies would benefit from pre-test measures or mediation analyses (e.g., correlating mindfulness adherence or self-report changes with extinction recall outcomes) to more firmly establish causal pathways. Sixth, the 6 mm FWHM smoothing kernel used in our analyses, while standard for 3T fMRI, may be suboptimal for capturing fine-grained subcortical and brainstem signals at 7T, where smaller kernels could better preserve spatial specificity. This may have reduced our sensitivity to detect small subcortical effects and should be considered in future 7T studies. Seventh, our sample size for brain-behavior correlations (*n* = 49) falls below the recommended threshold of 60 participants for reliable individual-difference analyses in neuroimaging, and therefore these exploratory correlational results should be interpreted with caution.

## Conclusion

This study provides preliminary neuroimaging evidence that mindfulness training enhances fear extinction recall, with observed effects in subcortical threat-processing regions. Using a readily accessible app-based intervention, our findings are consistent with the possibility that mindfulness may operate through implicit rather than explicit emotion regulation—a mechanism that, if confirmed in larger samples, may be particularly valuable for patients with limited cognitive control capacity or high emotional reactivity.

Our results suggest that pre-treatment mindfulness training could enhance exposure therapy outcomes by improving the neuroplastic foundation for extinction learning. By contributing to our understanding of how mindfulness may modulate fear memory circuits, this work connects contemplative neuroscience and clinical psychology. The use of a readily accessible app-based intervention suggests potential for translation to clinical practice, though replication in larger and clinical samples is needed before conclusions about clinical utility can be drawn.

## Data Availability

The datasets generated during and/or analyzed during the current study are available from the corresponding author on reasonable request.
